# A new year, a big year

**DOI:** 10.1111/iwj.14070

**Published:** 2022-12-28

**Authors:** Douglas Queen, Keith Harding

**Affiliations:** ^1^ Editor IWJ; ^2^ Editor‐in‐Chief IWJ

As the world enters 2023, COVID remains, and many health care systems are in crisis. Economic challenges impact many in the world, making it seem that the pandemic effect lingers and challenges humankind. Hopefully 2023 provides a better year and the opportunity for all involved to guide the world back to the “old normal”. While this seems unlikely at present, we must remain optimistic and use these experiences as a learning opportunity. This is true in wound care as well.

Wound care has for decades been a health care challenge. Up until the COVID pandemic, wound carers as a specialty, were winning the battle, addressing the many significant challenges faced by those who suffer from acute and chronic wounds. Yes, COVID stalled or reversed some of this progress. The biggest challenge during pandemic times was patient access to specialised care. To those of us who have been around wound care for several decades, this reflected the past. We were not alone, as many other clinical specialties suffered the same fate. One positive pandemic effect was the emergence of virtual visits. While these are not ideal for patients with wounds, they did present an opportunity. That opportunity was the support of more homecare based wound practitioners with more specialised resources. At home access to the specialist! Society learned to do things differently, proliferating practices that were being tested in the field prior to COVID, a pandemic acceleration perhaps. Some of this remains today and will endure into the future.

While 2022 was a challenging year, and perhaps 2023 will throw up new challenges, perhaps we need to look at these challenges as opportunities for change. The lack of resourcing, both human and financial, is not new to wound care, in fact, it has been a constant for as long as most wound carers can remember. Therefore, 2023 could be the year of opportunity to bring technological solutions to the forefront for the management of persons with wounds. A time to show governments and other payers that technological solutions can drive efficiency in a resource strapped environment. Governments and health systems around the world are in crisis management mode. During this time, they are perhaps more willing to listen to any solution that provides efficiency in such a crisis.

As wound carers it is time for us to push technologies that provide any efficiency in the management of persons with wounds. It is time to present solutions rather than highlight problems. Make 2023 a year that counts in the continued evolution of wound care as a specialty. Make 2023 a major step in winning the war against wounds.

Happy New Year to all of our readers and contributors.

